# Association between United States Environmental Contaminants and the Prevalence of Psoriasis Derived from the National Health and Nutrition Examination Survey

**DOI:** 10.3390/toxics12070522

**Published:** 2024-07-19

**Authors:** Linfen Guo, Beilin Tu, Deng Li, Lin Zhi, Yange Zhang, Haitao Xiao, Wei Li, Xuewen Xu

**Affiliations:** Department of Plastic and Burns Surgery, West China Hospital, Sichuan University, Chengdu 610041, China

**Keywords:** psoriasis, environmental contaminants, NHANES, WQS regression

## Abstract

(1) Background: Prolonged coexposure to environmental contaminants is reportedly associated with adverse impacts on skin health. However, the collective effects of contaminant mixtures on psoriasis prevalence remain unclear. (2) Methods: A nationally representative cohort study was conducted using data from the National Health and Nutrition Examination Survey 2003–2006 and 2009–2014. The association between contaminant exposures and psoriasis prevalence was analyzed through weighted quantile sum regressions, restricted cubic splines, and multivariable logistic regression. (3) Results: 16,453 participants and 60 contaminants in 8 groups were involved. After adjusting for demographics and comorbidities, exposure to urinary perchlorate, nitrate, and thiocyanate mixtures (OR: 1.10, 95% CI: 1.00–1.21) demonstrated a significant positive linear association with psoriasis prevalence. Ethyl paraben (OR: 1.21, 95% CI: 1.02–1.44) exhibited a significant positive correlation with psoriasis risk as an individual contaminant. The association between blood cadmium, lead, and mercury mixtures (OR: 1.10, 95% CI: 1.00–1.21), urinary perchlorate, nitrate, and thiocyanate mixtures (OR: 1.16, 95% CI: 1.00–1.34), and psoriasis prevalence was more pronounced in the lower healthy lifestyle score subgroup. (4) Conclusions: Exposure to perchlorate, nitrate, and thiocyanate mixtures, and ethyl paraben was associated with an elevated psoriasis prevalence. Furthermore, the association between cadmium and lead and mercury mixtures as well as perchlorate, nitrate and thiocyanate mixtures, and psoriasis prevalence was more pronounced in individuals with less healthy lifestyles.

## 1. Introduction

Psoriasis, a chronic and immune-mediated cutaneous condition, is commonly characterized as the presence of widespread erythematous plaques covered with a silvery-white scale [1]. This prevalent condition affects over 7.5 million adults, accounting for approximately 3.0% of the US adult population [2]. Males exhibited an estimated twofold higher susceptibility to psoriasis compared to females [3,4]. Psoriasis prevalence has been gradually increasing, impairing patients’ life quality, treatment adherence, and therapeutic satisfaction and leading to a remarkable socioeconomic burden [5,6]. Evidence indicates the existence of intricate interactions among gene variants and environment exposures in the expression of psoriasis [7]. Recently, psoriasis has been recognized as a systemically inflammatory disease that potentially leads to the progression of obesity, arthritis, hypertension, diabetes, and cardiovascular diseases (CVDs) [8]. Moreover, psoriasis is strongly linked to unhealthy lifestyles such as smoking [9,10], excessive alcohol consumption [11,12], imbalanced dietary habits [13,14], and insufficient physical activity [15].

With the escalating industrialization, numerous chemical contaminants are released into the environment. Prolonged coexposure to these contaminants has been associated with adverse impacts on skin health such as premature aging, photodamage, solar lentigines and melasma, and trigger acne vulgaris, pathologic pigmentation, atopic dermatitis, and psoriatic lesions [16,17,18]. For example, experimental evidence indicates that ozone exposure positively correlates with oxidative damage, compromised antioxidant defense, and a proinflammatory response in the skin [16,19]. Epidemiological studies identified a direct association between airborne particulate matter exposure and the manifestation of skin photoaging signs, particularly hyperpigmented lesions and wrinkles [20]. In addition, exposure to particulate matter diameter < 2.5 µm (PM2.5) has been significantly associated with the development of solar lentigines on the cheeks and the backs of hands [21]. Notably, some contaminants, such as polycyclic aromatic hydrocarbons, exhibit photoreactive and phototoxic properties, particularly in the presence of ultraviolet A [22]. Contaminants and sunlight might thus synergistically exacerbate skin damage [23]. In addition, a previous investigation showed that individuals with severe psoriasis exhibited elevated levels of blood cadmium, and environmental cadmium exposure possibly contributed to the exacerbation of psoriasis [24].

Considering that the coexistence of various contaminants potentially leads to additive, antagonistic, synergistic, or other complex interactions, relying solely on the effects of an individual contaminant may not provide a comprehensive understanding of the pathogenetic mechanisms of psoriasis. However, current evidence regarding the association between environmental contaminant mixtures and psoriasis development remains relatively scarce. An epidemiological investigation explored the positive correlation between exposure to large-scale air pollutant mixtures and diagnosed psoriasis, which was not observed in single-pollutant models [25]. However, this study specifically targeted common air pollutants such as PM2.5 and BTEX contaminants (benzene, toluene, ethylbenzene, and xylene). A recent nationwide study exclusively focused on the link between mixed or individual environmental metals and psoriasis prevalence based on the NHANES population [26]. In addition to environmental metals, arsenic species, dioxins, and PAHs have also been investigated in relation to skin conditions [27,28,29,30]. However, the majority of relevant studies were generally conducted with relatively small sample sizes and did not consistently focus on psoriasis prevalence as the primary outcome. Furthermore, some contaminants like phthalates and polyfluoroalkyl chemicals were hardly assessed in former investigations. To address the aforementioned gap, we intended to investigate the simultaneous association between commonly encountered environmental contaminants across 8 chemical groups (in total 60 chemical substances) and psoriasis prevalence in the National Health and Nutrition Examination Survey (NHANES) [31,32].

## 2. Materials and Methods

### 2.1. Research Population

**Figure 1:**
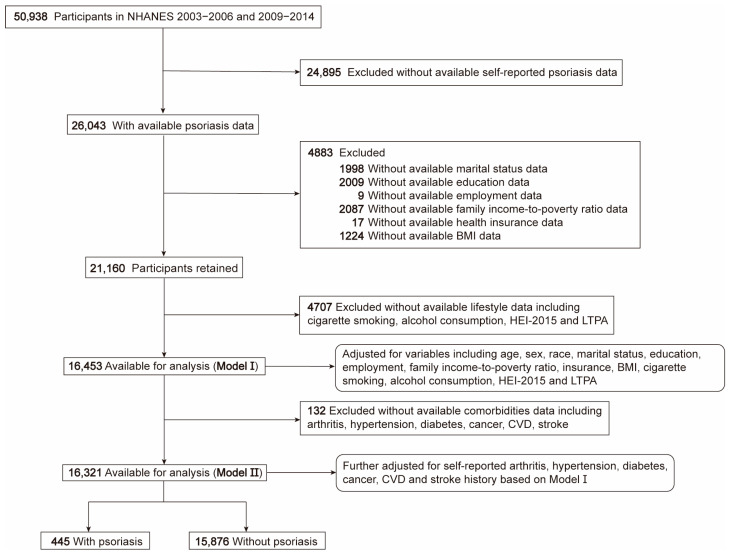
Flow chart of participants who met inclusion criteria and were included in this study. Model I was adjusted for variables including age, sex, race, marital status, education, employment, family income-to-poverty ratio, health insurance, BMI, cigarette smoking, alcohol consumption, HEI-2015, and LTPA. Model II was further adjusted for self-reported arthritis, hypertension, diabetes, cancer, CVD, and stroke history based on Model I. BMI, body mass index; CVD, cardiovascular disease; HEI, healthy eating index; LTPA, leisure time physical activity.

The NHANES is a continuous and nationwide cross-sectional survey program with rigorous stratified and clustered sampling techniques implemented by the National Center for Health Statistics (NCHS) to assess the health and nutritional status of noninstitutionalized civilians in the United States. The NHANES data consist of interview, examination, and physiological measurement components, making it a valuable resource for large-scale epidemiological studies and health science researchers. Data on individuals from NHANES cycles of 2003–2006 and 2009–2014 were utilized. We sequentially excluded those without available information on self-reported psoriasis history, demographics, lifestyles, and comorbidities (Figure 1). The number of participants identified for each of the 8 contaminant mixture groups varied across different survey cycles considering the data availability of respective variables (Table S1). The ethics approval for NHANES had been obtained from the NCHS ethic review board. And all methods were performed in accordance with the relevant NHANES guidelines and regulations. We sequentially excluded subjects lacking self-reported psoriasis history, demographic data, lifestyle data, and comorbidity data.

### 2.2. Psoriasis Definition

Consistent with the methodology employed in prior epidemiological investigations, psoriasis was defined as a positive response to the inquiry, “Have you ever been told by a doctor or other health care provider that you had psoriasis?” in dermatology or medical condition questionnaires [33,34]. A total of 26,043 participants responding to this psoriasis survey question proceeded to the next round of participant screening.

### 2.3. Environmental Contaminant Exposures

The degree of exposure to environmental contaminants was assessed through the measurement of chemical concentrations in blood or urine biological samples of the participants. We determined the environmental contaminants involved in this study based on data completeness and study interest, and individual contaminants in categories missing in 80% of the total samples were not considered for further analysis. Ultimately, we identified 60 exposure analytes categorized into 8 mixture groups including arsenic species in urine (*n* = 7), cadmium, lead and mercury in blood (*n* = 3), perchlorate, nitrate, and thiocyanate in urine (*n* = 3), phenols in urine (*n* = 7), phthalates in urine (*n* = 13), polyaromatic hydrocarbons in urine (PAHs, *n* = 9), polyfluoroalkyl chemicals in blood (*n* = 12), and pesticides in urine (*n* = 6) (specific chemicals of environmental contaminants in different mixture groups were present in Table S1). Notably, measured concentrations of contaminants lower than the lowest limit of detection (LLOD) were substituted with LLOD/√2 according to the analysis guide of the NHANES database. Owing to varying sampling ratios of specific contaminants across different survey cycles, the number of participants examined for contaminants differed, with the Model I cohort ranging from 3372 to 13,772 individuals and the Model II cohort ranging from 3341 to 13,659 individuals across different contaminants as presented in Table 1 and Table 2. The Model I cohort was adjusted for covariates including age, sex, race, marital status, education, employment, family income-to-poverty ratio, health insurance, body mass index (BMI), and lifestyle factors composed of cigarette smoking, alcohol consumption, diet quality assessed by healthy eating index scores-2015 (HEI-2015), and leisure time physical activity (LTPA). Model II was a fully adjusted model that additionally accounted for self-reported comorbidities including arthritis, hypertension, diabetes, cancers, CVDs, and stroke. The measurement, calibration, and result interpretation for contaminant chemicals were processed based on the NHANES Laboratory/Medical Technologists Procedures Manual (e.g., blood cadmium, lead, and mercury, https://wwwn.cdc.gov/nchs/data/nhanes/2003-2004/labmethods/l06_c_met_pb_cd_hg.pdf (accessed on 14 June 2024)).

### 2.4. Covariates

The current study examined four factors related to healthy lifestyles, namely cigarette smoking, alcohol consumption, diet quality, and LTPA. Details on how these variables were calculated were available in the associated published study [31]. Briefly, smoking intensity was determined through the daily smoking amount from self-reported questionnaires [32]. Consumed alcoholic amount was quantified as average drinks per day (one drink approximating a 12-ounce beer, 5-ounce glass of wine, or 1.5-ounce shot of liquor) based on a self-reported quantity of alcoholic consumption within the past year [35]. Dietary quality was evaluated through HEI-2015, which incorporated information from 24-h diet recall and analysis of nutrient consumption utilizing the Nutrient Database for Dietary Studies and Food Patterns Equivalents Database of the US Agriculture Department [36]. Lastly, LTPA was assessed using metabolic equivalent times (METs), which combined activity duration in minutes and matched metabolic equivalent scores [37]. The judging criteria for a healthier lifestyle consisted of no cigarette smoking, alcohol consumption below the recommended limit according to the dietary guidelines for Americans, and an HEI score or LTPA above the median [38]. The calculation of the healthy lifestyle score (HLS) involved summing the points allocated to each indicator, marking 1 point if participants fulfilled any of the judging criteria. The HLS ranged from 0 to 4, and higher scores denoted a healthier lifestyle [31]. Additionally, other covariates considered in the analysis involved age, sex, race, marital status, education, employment, family income-to-poverty ratio, health insurance, BMI as well as previously diagnosed comorbidities (arthritis, hypertension, diabetes mellitus, cancers, CVDs, and stroke).

### 2.5. Statistical Analysis

The demographic characteristics of the recruited individuals were described as mean and standard deviation (SD) for continuous variables and percentage frequencies for categorical variables. Continuous variables were compared by Kruskal–Wallis rank sum tests considering not satisfying normality and categorical variables were compared utilizing the χ^2^ tests.

The association between environmental contaminant mixture groups and the prevalence of psoriasis was assessed using weighted quantile sum (WQS) regression, implemented with the R package “Gwqs”. WQS regression, a principle component analysis approach, achieved this objective by incorporating selection and dimensionality reduction features through the calculation of the WQS index. Two different WQS models were utilized, adjusting for various potentially confounding factors. The major contributing contaminant factor in each group to psoriasis risk could be identified easily by comparing corresponding weights in the WQS regression results. The weights of individual environmental contaminants spanned a range of 0 to 1, where higher weights demonstrated a greater degree of importance. To perform the WQS analyses, the dataset was separated into training and validation sets in a randomized manner, with a ratio of 40:60. The weights of contaminants were obtained through 200 bootstrap iterations performed on the training dataset, while the validation dataset was employed to assess the significance of these contaminants. In particular, the overall contaminant exposures were constrained to act in a consistent direction to avoid the reversal paradox, supposed to be positive in the current study. Moreover, restricted cubic splines (RCSs) were employed to explore potential nonlinear relationships between WQS indexes of the 8 environmental contaminant mixture groups and psoriasis prevalence while controlling for the aforementioned confounding variables. Optimal knots were selected using the Akaike information criterion with the smallest value and nonlinearity tests were conducted utilizing the “ANOVA” function within the “RMS” package to determine the potential dose-dependent correlations. Furthermore, subgroup analyses were performed to investigate whether the connection between contaminant mixture groups and psoriasis incidence varied based on the combined influence of the four lifestyle factors. All final results of the WQS regressions were reported as odds ratios (ORs) along with respective 95% confidence intervals (CIs).

Multivariable logistic regression analysis was employed to explore the links between individual contaminants and psoriasis prevalence by two models, adjusting the above-mentioned confounding factors in WQS regression. Given the right-skewed distribution of chemical contaminant concentrations in participants’ blood and urine, corresponding data were subject to In-transformation to approximate the normal distribution. The NHANES analytic guidelines were followed to incorporate sample weights across different study cycles and the results were presented as ORs and respective 95% CIs as well. Statistical analyses were performed through R software, version 4.2.2. The two-side *p*-values below 0.05 were deemed statistically significant.

## 3. Results

### 3.1. Characteristics of the Participants

A total of 16,453 individuals aged 20–80 were involved in Model I, 455 of whom had been previously diagnosed with psoriasis. Owing to missing comorbidities data, 132 participants were excluded from Model II. The baseline demographic information is presented in Table S2. Participants diagnosed with psoriasis, accounting for 2.7% of the total population, were found to be significantly older (*p* < 0.001), exhibited a greater likelihood of unemployment (*p* = 0.008), had higher BMIs (*p* < 0.001), smoked more (*p* < 0.001), engaged in less physical activity (*p* = 0.016), and were linked to a higher prevalence of comorbidities involving arthritis (*p* < 0.001), hypertension (*p* < 0.001), diabetes (*p* = 0.002), cancer (*p* < 0.001), and CVD (*p* < 0.001). Additionally, non-Hispanic white individuals exhibited a stronger association with a history of psoriasis (*p* < 0.001). The specific number of subjects in each contaminant mixture group is presented in Table 1.

### 3.2. Association between Contaminant Exposure and Psoriasis Prevalence

**Figure 2:**
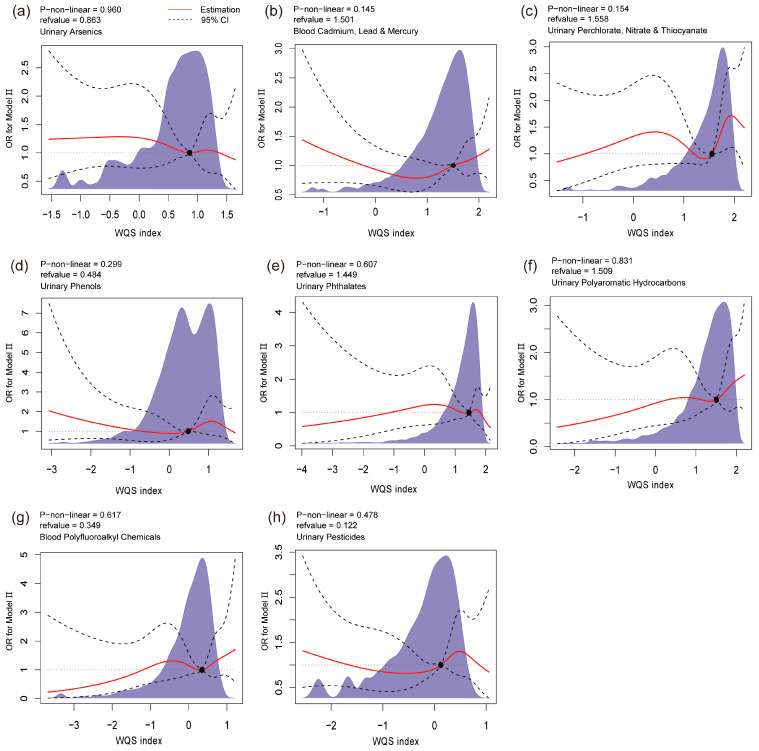
Restricted spline curves for correlations between environmental contaminant mixture groups (WQS indexes) and psoriasis prevalence (Model II cohort). (**a**) Arsenic species in urine; (**b**) cadmium, lead, and mercury in blood; (**c**) perchlorate, nitrate, and thiocyanate in urine; (**d**) phenols in urine; (**e**) phthalates in urine; (**f**) polyaromatic hydrocarbons in urine; (**g**) polyfluoroalkyl chemicals in blood; (**h**) pesticides in urine. The shaded areas depict data distribution, while the dotted lines correspond to the 95% confidence intervals. Model II was adjusted for age, sex, race, marital status, education, employment, family income-to-poverty ratio, health insurance, BMI, cigarette smoking, alcohol consumption, HEI-2015, LTPA, and self-reported history of arthritis, hypertension, diabetes, cancer, cardiovascular disease, and stroke.

We conducted WQS regression to explore the combined role of contaminant mixture exposures on psoriasis prevalence. As shown in Table 1, urinary perchlorate, nitrate, and thiocyanate as a group demonstrated a significant positive association with psoriasis prevalence in the Model II cohort (OR: 1.10, 95% CI: 1.00–1.21). However, such a correlation did not appear in the same mixture group of the Model I cohort, nor the other 7 contaminant mixture groups of either the Model I cohort and Model II cohort. The respective weights of each component in contaminant mixtures were presented in Figures S1 and S2. In addition, we utilized RCS curves to assess the potential nonlinear relationship in Figure 2 and Figure S3. No potential nonlinear relationships between any contaminant mixtures and psoriasis prevalence were observed in either the Model I or Model II cohort.

Additionally, we selected the three components of the urinary perchlorate, nitrate, and thiocyanate mixture group exhibiting statistical significance in WQS regressions as well as the individual contaminant occupying the highest weight of other seven mixture groups in both cohorts. Subsequently, we investigated the associations between these selected contaminants and psoriasis prevalence by multivariate logistic regression analysis (Table 2). In both cohorts, the majority of contaminants did not significantly and positively associate with psoriasis occurrence, including the three components of urinary perchlorate, nitrate, and thiocyanate mixture group. Notably, ethyl paraben in the urinary phenols exhibited a significant positive association with psoriasis prevalence in the Model I cohort (OR: 1.21, 95% CI: 1.02–1.44) and an approximately significant positive association in the Model II cohort (OR: 1.19, 95% CI: 1.00–1.43) as an individual contaminant.

### 3.3. WQS Regressions Stratified by Healthy Lifestyle Score

WQS regressions were further stratified by HLS and the majority of contaminant mixture groups continued to not display significant associations in various HLS subgroups (Table 3). Subgroup analysis did not provide clear evidence of lifestyle factors modifying the combined effect for most of the contaminant mixture groups in the multiplicative scale (*p* > 0.05). However, mixture groups of urinary perchlorate, nitrate, and thiocyanate, which exhibited a significantly positive connection with psoriasis prevalence in the Model II cohort of unstratified WQS regression, showed a similar association in the HLS 0–2 subgroup of the Model I cohort (OR: 1.16, 95% CI: 1.00–1.34). Additionally, the mixture group of blood cadmium, lead, and mercury displayed an approximately significant association with psoriasis prevalence in the HLS 0–2 subgroup of the Model I cohort as well (OR: 1.10, 95% CI: 1.00–1.21).

## 4. Discussion

The present study represented a comprehensive investigation into the simultaneous association between common environmental contaminants and the risk of psoriasis in a nationally representative population. After adjusting for demographics and comorbidities, exposure to urinary perchlorate, nitrate, and thiocyanate mixtures demonstrated a significant positive linear association with psoriasis prevalence. Ethyl paraben in the urinary phenols exhibited a significant positive correlation with psoriasis risk as an individual contaminant. In addition, the association between blood cadmium, lead, and mercury mixtures, as well as urinary perchlorate, nitrate, and thiocyanate mixtures and psoriasis prevalence were more pronounced in the lower HLS subgroup, indicating the mediating role of lifestyles. These results shed light on the possible influence of prolonged environmental contaminant exposure on the onset and development of psoriasis.

Chronic arsenic exposure has been illustrated as resulting in neuropathic itch by β-endorphin, which was commonly elevated in atopic dermatitis and psoriasis [39]. Significant differences in cadmium levels were observed between participants with psoriasis and those without, and higher total concentrations of cadmium, lead, chromium, and nickel have been found in individuals with psoriasis living near cement factories [28,40]. A similar study further revealed that the concentration of cadmium was connected with the severity degree of psoriasis [41]. However, considering the low-dose exposures of environmental contaminants in NHANES participants and complex interactions among various chemicals of the same group, our study did not reveal a significant combined connection between cadmium, lead, mercury, or various arsenic species and the occurrence of psoriasis. Moreover, previous studies have indicated that the link between psoriasis risk and environmental contaminants could be more pronounced among individuals with severe psoriasis, thus overlooking the severity assessment might lead to an underestimation of this connection [24,41]. Another potential explanation was that the different types of chemical contaminants investigated in this study were exclusively assessed in either blood or urine samples given the data availability, and these biological substrates possessed distinct metabolic and excretory toxicological properties [42]. A recent study supporting this notion revealed a contrasting finding, where significant correlations were found between urinary metal mixtures and the risk of psoriasis, while the metal mixtures in blood did not reach a significantly positive level [26]. Therefore, more relevant epidemiological studies utilizing biological samples from diverse sources are still necessary to validate and reinforce these findings.

Notably, limited studies have explored the correlations between contaminant exposure and psoriasis prevalence, aside from studies focused on air pollutants [16]. Exposure to air pollutants such as PAHs, particulate matter, and volatile organic compounds activates the aryl hydrocarbon receptor and further activates Th17 cells, serving as the most critical cells implicated in the pathogenetic mechanism of psoriasis [43]. Four large-scale studies have demonstrated a significant association between short-term exposure to air pollutants and an elevated incidence of outpatient visits for psoriasis lesions [44,45,46,47]. These studies collectively underscored the necessity of considering environmental contaminants in the management of psoriasis. However, contaminants arising from drinking water, diet, and even occupational exposure should not be overlooked as well, thus this study emphasizes the investigation of ubiquitous environmental contaminants encompassing a wide range of potential sources. The limited number of previous investigations hinders a comprehensive elucidation of the mechanism underlying the association between environmental contaminant mixtures and psoriasis risk in our study. However, this also highlights the novelty and originality of our research.

Considering the moderating impact of lifestyles on environmental toxicity, it is recommended to include lifestyle factors when analyzing the damage caused by environmental contaminants and the associated disease risks [48]. For instance, potential adverse health effects associated with exposure to polychlorinated biphenyls may increase with the ingestion of certain dietary fats, whereas the consumption of fruits and vegetables, which are rich in antioxidant and anti-inflammatory nutrients or bioactive compounds, may offer protective benefits [48]. Cigarette smoking markedly increases environmental exposure to toxic contaminants such as nicotine, tar, phenols, alkanes, polycyclic aromatic hydrocarbons, heavy metals, and organic pesticides [49]. Moreover, recent research indicates that physical activity may mitigate health risks posed by exposure to environmental chemicals [50]. Therefore, we investigated the mediating role of lifestyles in the relationship between environmental contaminant exposure and psoriasis risk. Interestingly, our findings revealed that the association between cadmium, lead, mercury, as well as perchlorate, nitrate, thiocyanate, and psoriasis prevalence were more pronounced in the lower HLS subgroup, which implied that reducing contaminant exposures may yield greater benefits in averting the onset of psoriasis for individuals with less healthy lifestyles. It is plausible that the etiology of psoriasis may differ between individuals with unhealthy and healthy lifestyles. In addition, our findings in the Model I- and Model II-defined cohorts did not show total consistency, indicating that the comorbidity covariates involved in this study may exhibit a complex mediating role in the progression of psoriasis. Therefore, it is recommended that future relevant studies take into account lifestyle differences and strengthen the comprehension of the intricate interplay between comorbidities and psoriasis. Furthermore, psoriasis is a complex polygenic genetic disease that frequently exhibits familial aggregation [51]. Several gene loci responsible for psoriasis susceptibility have been identified and termed psoriasis susceptibility locus (PSORS) 1–10 [52]. The PSORS1 locus demonstrates the strongest association, accounting for 35–50% of disease heritability [53]. The HLA-Cw * 06 allele is particularly associated with the development of psoriasis, correlating with early onset and a severe disease course [54]. In genetically predisposed individuals, trigger factors such as alcohol consumption may contribute to the onset and exacerbation of symptoms [14]. Furthermore, while most studies indicate that the absolute magnitude of the difference between genders in psoriasis prevalence is minimal, no research has yet demonstrated that gender differences do not influence the relationship between environmental contaminants and psoriasis prevalence [7,55]. Thus, future relevant research is suggested to consider genetic and gender differences when exploring this relationship. Additionally, further research is necessary for the contaminants involved in this study not showing significant associations with psoriasis including phthalates, PAHs, polyfluoroalkyl chemicals, and pesticides. A point worth noting is that certain arsenic compounds and PAHs, such as arsenic trioxide and crude coal tar, have been utilized as feasible therapeutic agents for psoriasis. This means even medical therapies may contribute to adverse health impacts, such as the induction of oxidative stress and genotoxic damage [56]. Current understanding of the molecular mechanisms indicates that similar mechanisms mediate both the therapeutic activities and the toxic effects of arsenic species [27]. Exposures to PAHs during the therapy period are comparable to, and in some cases even slightly higher than, standard occupational exposures [30]. Therefore, the rational use of arsenic species and PAHs following the appropriate dosage is of substantial importance and more comprehensive and meticulous experimental assessments must be performed.

However, there were also several inevitable limitations worth noting. First, the cross-sectional design of the NHANES database restricted our ability to establish causal relationships between contaminant exposures and psoriasis prevalence. Second, the diagnosis of psoriasis depended on self-reported questionnaires rather than clinical diagnosis, which introduces the possibility of recall and information biases. Third, despite the data availability for the extent of psoriasis lesions in NHANES, the association between psoriasis severity and contaminant exposure was not explored due to limitations in the sample size of the psoriasis questionnaire and various chemical examinations in the blood and urine. Fourth, certain contaminants within each type were not involved in this study owing to data unavailability, which could potentially impact the investigation into the combined effect of each contaminant mixture group. Last, although we utilized cigarette smoking, alcohol consumption, diet quality, and physical activity to construct the HLS for overall lifestyle evaluation, the definition remains relatively ambiguous. Future investigations could benefit from a more comprehensive collection of lifestyle-related variables, potentially drawing from diverse databases or multiple large population cohorts [57].

In conclusion, the results of this nationally representative cohort study suggested that exposure to perchlorate, nitrate, and thiocyanate mixtures and ethyl paraben was associated with an elevated psoriasis prevalence. Furthermore, the association between cadmium, lead, and mercury mixtures as well as perchlorate, nitrate, and thiocyanate mixtures and psoriasis prevalence were more pronounced in individuals with less healthy lifestyles. Further prospective studies are warranted to validate these observations.

## Figures and Tables

**Table 1:** Weighted quantile sum regressions recognizing contaminant mixture exposure factors related to psoriasis prevalence.

Variables	Model I ^1^			Model II ^2^		
	Total/Psoriasis ^3^	OR (95%CI)	*p* Value	Total/Psoriasis	OR (95%CI)	*p* Value
Urinary arsenic species	5381/149	0.97 (0.81, 1.15)	0.721	5345/145	0.90 (0.75, 1.08)	0.244
Blood cadmium, lead, and mercury	13,772/382	0.97 (0.89, 1.05)	0.413	13,659/374	1.04 (0.96, 1.13)	0.305
Urinary perchlorate, nitrate, and thiocyanate	6086/166	1.03 (0.95, 1.13)	0.464	**6047/161**	**1.10 (1.00, 1.21)**	**0.049**
Urinary phenols	3372/97	1.09 (0.89, 1.34)	0.408	3341/95	1.08 (0.88, 1.33)	0.471
Urinary phthalates	3372/97	0.95 (0.83, 1.10)	0.507	3341/95	0.99 (0.87, 1.13)	0.888
Urinary polyaromatic hydrocarbons	3755/105	1.03 (0.93, 1.15)	0.585	3717/103	1.01 (0.91, 1.13)	0.840
Blood polyfluoroalkyl chemicals	3664/97	1.08 (0.73, 1.61)	0.690	3629/95	1.00 (0.68, 1.47)	0.992
Urinary pesticides	3967/104	1.11 (0.73, 1.68)	0.635	3929/104	0.95 (0.65, 1.40)	0.799

OR, odds ratio; CI, confidence interval. Significant values are in bold. ^1^ Model I was adjusted for variables including age, sex, race, marital status, education, employment, family income-to-poverty ratio, health insurance, BMI, cigarette smoking, alcohol consumption, HEI-2015, and LTPA. ^2^ Model II was further adjusted for self-reported arthritis, hypertension, diabetes, cancer, cardiovascular disease, and stroke history based on Model I. ^3^ Total/Psoriasis, total number of participants/number of participants diagnosed with psoriasis.

**Table 2:** Multivariate logistic regression recognizing individual contaminant exposure factors related to psoriasis prevalence.

Model I ^1^			Model II ^2^		
Variables	OR (95%CI)	*p* Value	Variables	OR (95%CI)	*p* Value
Urinary perchlorate, nitrate, and thiocyanate			Urinary perchlorate, nitrate, and thiocyanate		
Thiocyanate (ng/mL)	1.06 (0.89, 1.28)	0.508	Thiocyanate (ng/mL)	1.08 (0.89, 1.31)	0.426
Perchlorate (ng/mL)	1.02 (0.82, 1.27)	0.868	Perchlorate (ng/mL)	1.05 (0.83, 1.33)	0.710
Nitrate (ng/mL)	0.94 (0.76, 1.16)	0.566	Nitrate (ng/mL)	0.95 (0.75, 1.21)	0.683
Urinary arsenic species			Urinary arsenic species		
Arsenic acid (ug/L)	0.96 (0.44, 2.11)	0.923	Arsenic acid (ug/L)	0.99 (0.46, 2.11)	0.974
Blood cadmium, lead, and mercury			Blood cadmium, lead, and mercury		
Cadmium (ug/L)	0.95 (0.79, 1.12)	0.519	Cadmium (ug/L)	0.92 (0.78, 1.09)	0.331
Urinary phenols			Urinary phenols		
**Ethyl paraben** **(ng/mL)**	**1.21 (1.02, 1.44)**	**0.041**	**Ethyl paraben (ng/mL)**	**1.19 (1.00, 1.43)**	**0.053**
Urinary phthalates			Urinary phthalates		
Mono-benzyl phthalate (ng/mL)	1.04 (0.86, 1.24)	0.716	Mono-(2-ethyl-5-oxohexl) phthalate (ng/mL)	1.01 (0.87, 1.18)	0.907
Urinary polyaromatic hydrocarbons			Urinary polyaromatic hydrocarbons		
2-hydroxynaphthalene (ng/L)	1.15 (0.95, 1.40)	0.165	1-hydroxynaphthalene (ng/L)	0.99 (0.87, 1.12)	0.824
Blood polyfluoroalkyl chemicals			Blood polyfluoroalkyl chemicals		
Perëuoroheptanoic acid (ng/mL)	1.11 (0.79, 1.55)	0.545	Perëuoroheptanoic acid (ng/mL)	1.06 (0.73, 1.52)	0.759
Urinary pesticides			Urinary pesticides		
2-isopropyl-4-methylpyrimidinol (ug/L)	0.79 (0.52, 1.20)	0.260	2-isopropyl-4-methylpyrimidinol (ug/L)	0.80 (0.53, 1.22)	0.288

OR, odds ratio; CI, confidence interval. Significant values are in bold. ^1^ Model I was adjusted for variables including age, sex, race, marital status, education, employment, family income-to-poverty ratio, health insurance, BMI, cigarette smoking, alcohol consumption, HEI-2015, and LTPA. ^2^ Model II was further adjusted for self-reported arthritis, hypertension, diabetes, cancer, cardiovascular disease, and stroke history based on Model I.

**Table 3:** Weighted quantile sum regressions recognizing contaminant mixture exposure factors related to psoriasis prevalence in stratified analysis by HLS.

Variables	HLS 0–2 (Model I ^1^)			HLS 3–4 (Model I)			HLS 0–2 (Model II ^2^)			HLS 3–4 (Model II)		
	Total/ Psoriasis ^3^	OR (95%CI)	*p* Value	Total/ Psoriasis	OR (95%CI)	*p* Value	Total/ Psoriasis	OR (95%CI)	*p* Value	Total/ Psoriasis	OR (95%CI)	*p* Value
Urinary arsenic species	2602/81	0.91 (0.66, 1.26)	0.571	2779/68	1.05 (0.77, 1.44)	0.754	2580/78	0.82 (0.60, 1.13)	0.233	2765/67	1.10 (0.82, 1.49)	0.529
Blood cadmium, lead, and mercury	**6754/211**	**1.10 (1.00, 1, 21)**	**0.059**	7018/171	0.93 (0.82, 1.04)	0.212	6685/204	1.03 (0.93, 1.14)	0.558	6974/170	0.95 (0.85, 1.06)	0.347
Urinary perchlorate, nitrate, and thiocyanate	**2957/89**	**1.16 (1.00, 1, 34)**	**0.049**	3129/77	0.97 (0.86, 1.09)	0.597	2933/85	1.08 (0.94, 1.24)	0.255	3114/76	0.93 (0.81, 1.06)	0.254
Urinary phenols	1651/53	1.14 (0.84, 1.55)	0.395	1721/44	1.20 (0.91, 1.59)	0.203	1628/51	1.19 (0.88, 1.60)	0.255	1713/44	1.16 (0.91, 1.49)	0.237
Urinary phthalates	1651/53	0.92 (0.77, 1.10)	0.353	1721/44	0.94 (0.75, 1.18)	0.603	1628/51	0.93 (0.75, 1.17)	0.553	1713/44	1.03 (0.81, 1.29)	0.834
Urinary polyaromatic hydrocarbons	1846/60	1.10 (0.96, 1.25)	0.182	1909/45	0.84 (0.69, 1.03)	0.102	1818/58	1.10 (0.95, 1.28)	0.219	1899/45	0.96 (0.82, 1.11)	0.565
Blood polyfluoroalkyl chemicals	1777/51	1.19 (0.72, 1.97)	0.499	1887/46	0.92 (0.46, 1.84)	0.818	1757/50	0.81 (0.41, 1.58)	0.534	1872/45	1.58 (0.69, 3.59)	0.279
Urinary pesticides	1947/54	0.76 (0.40, 1.46)	0.409	2020/50	1.45 (0.83, 2.54)	0.188	1925/54	0.79 (0.39, 1.56)	0.491	2004/50	1.05 (0.66, 1.66)	0.850

OR, odds ratio; CI, confidence interval. Significant values are in bold. ^1^ Model I was adjusted for variables including age, sex, race, marital status, education, employment, family income-to-poverty ratio, health insurance, BMI, cigarette smoking, alcohol consumption, HEI-2015, and LTPA. ^2^ Model II was further adjusted for self-reported arthritis, hypertension, diabetes, cancer, cardiovascular disease, and stroke history based on Model I. ^3^ Total/Psoriasis, total number of participants/number of participants diagnosed with psoriasis.

## Data Availability

The National Health and Nutrition Examination Survey data are publicly available at https://www.cdc.gov/nchs/nhanes/index.htm (accessed on 14 June 2024).

## Supplementary Materials

The following supporting information can be downloaded at: https://www.mdpi.com/article/10.3390/toxics12070522/s1, Figure S1: WQS index weights for the indexes associated with psoriasis prevalence in the Model I cohort; Figure S2: WQS index weights for the indexes associated with psoriasis prevalence in the Model II cohort; Figure S3: Restricted spline curves for correlations between environmental contaminant mixture groups (WQS indexes) and psoriasis prevalence (Model I cohort); Table S1: Types of environmental contaminant mixture groups included in the weighted quantile sum regressions; Table S2: Characteristics of participants in Model II cohort from NHANES 2003–2006 and 2009–2014 cycles.
