# Supplementary material for: Association between United States Environmental Contaminants and the Prevalence of Psoriasis Derived from the National Health and Nutrition Examination Survey
Source: Toxics. 2024 Jul 19;12(7):522. doi: 10.3390/toxics12070522 (PMC11281726; doi:10.3390/toxics12070522)
Supplement: Supplementary file 1 [file toxics-12-00522-s001.zip › toxics-3082011-supplementary.pdf]

Association between United States environmental contaminants and the prevalence of psoriasis derived from the National Health and Nutrition Examination Survey

Linfen Guo [1] , Beilin Tu [1] , Deng Li [1] , Lin Zhi [1] , Yange Zhang [1] , Haitao Xiao [1] , Wei Li [1,*] and Xuewen Xu [1,*]

[1] Department of Plastic and Burns Surgery, West China Hospital, Sichuan University, Chengdu 610041, China

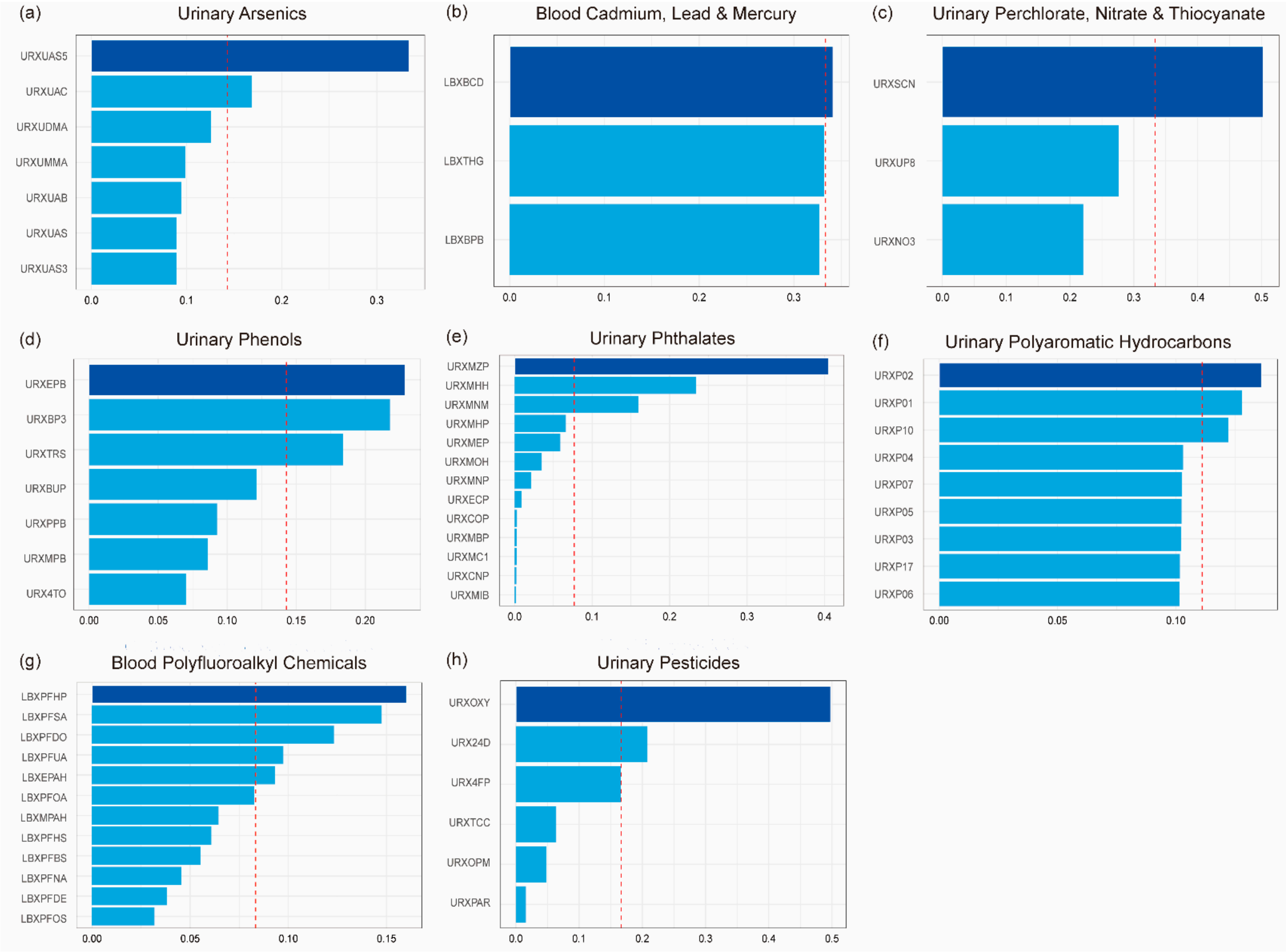

Figure S1 WQS index weights for the indexes associated with psoriasis prevalence in the Model I cohort. Contaminants with statistically significant weights within contaminant mixture groups included: (a) URXUAS5 (arsenic acid) and URXUAC (arsenocholine) within urinary arsenic species; (b) LBXBCD (cadmium) within Blood cadmium, lead and mercury; (c) URXSCN (thiocyanate) within urinary perchlorate, nitrate and thiocyanate; (d) URXEPB (ethyl paraben), URXBP3 (urinary 2-hydroxy-4-metoxybenzophenone(benzophenone-3)) and URXTRS (urinary 2,4,4'-Trichloro-2'-hydroxyphenyl ether (triclosan)) within urinary phenols; (e) URXMZP (mono-benzyl phthalate), URXMHH (mono-(2-ethyl-5-hydroxyhexyl)phthalate), URXMNM (mono-n-methyl phthalate) within urinary phthalates; (f) URXP02 (2-hydroxynaphthalene) and URXP01 (1-hydroxynaphthalene) , URXP10 (1-hydroxypyrene) within urinary polyaromatic hydrocarbons; (g) LBXPFHP (perëuoroheptanoic acid), LBXPFSA (perëuorooctane sulfonamide), LBXPFDO (perëurododecanoic acid), LBXPFUA (perëuoroundecanoic acid) and LBXEPAH (2-(n-ethyl-perëuorooctane sulfonamido) acetic acid) within blood polyfluoroalkyl chemicals; (h) URXOXY (oxypyrimidine) and URX24D (2,4-D) within Urinary pesticides. The remaining chemical contaminants represented by codes on this table could be found on the official website of NHANES.

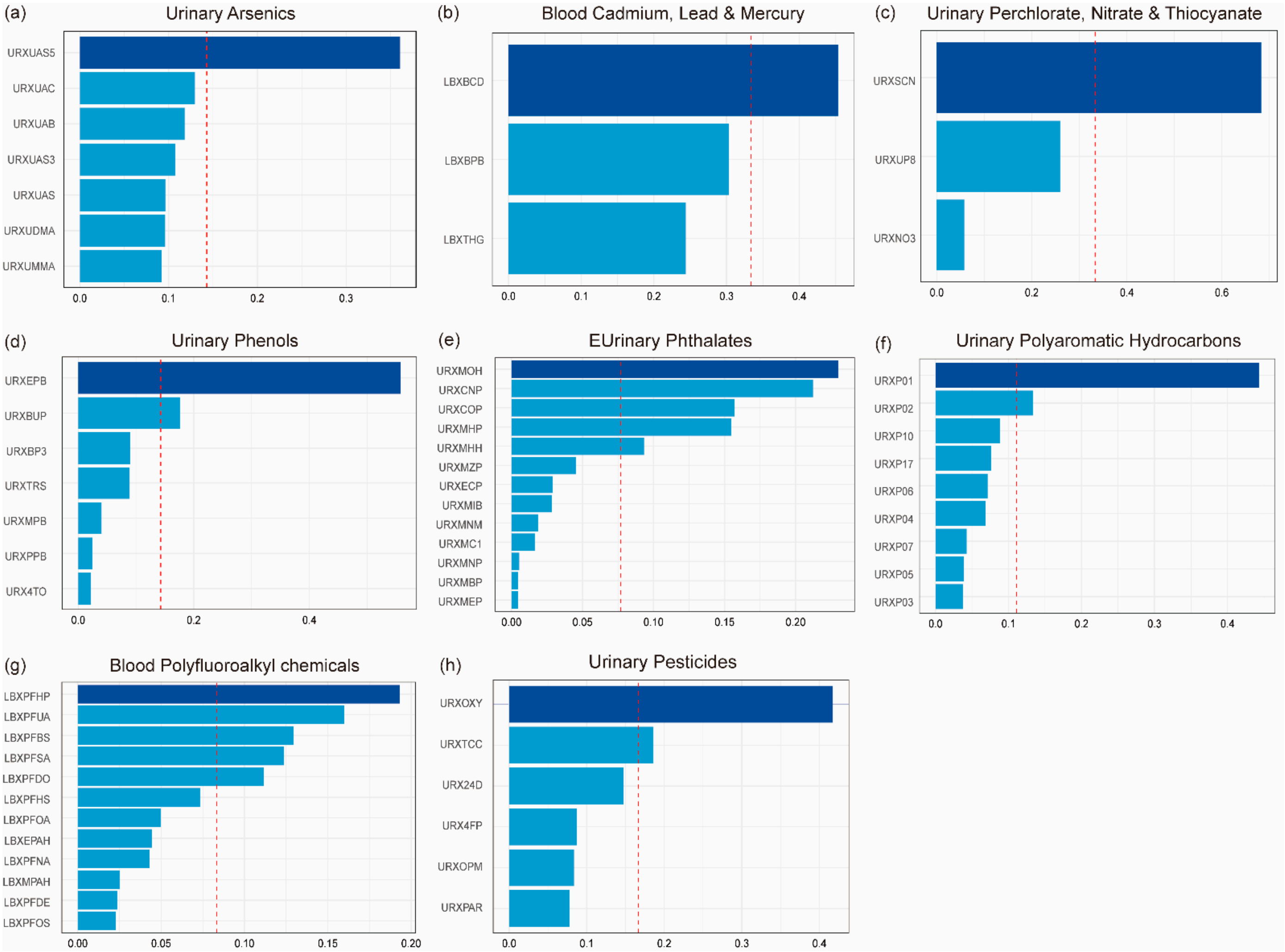

Figure S2 WQS index weights for the indexes associated with psoriasis prevalence in the Model II cohort. Contaminants with statistically significant weights within contaminant mixture groups included: (a) URXUAS5 (arsenic acid) within urinary arsenic species; (b) LBXBCD (cadmium) within Blood cadmium, lead and mercury; (c) URXSCN (thiocyanate) within urinary perchlorate, nitrate and thiocyanate; (d) URXEPB (ethyl paraben) and URXBUP (butyl paraben) within urinary phenols; (e) URXMOH (mono-(2-ethyl-5-oxohexyl) phthalate), URXCNP (mono(carboxynonyl) phthalate), URXCOP (Mono(carboxyoctyl) phthalate), URXMHP (mono-(2-ethyl)-hexyl phthalate) and URXMHH (mono-(2-ethyl-5-hydroxyhexyl) phthalate) within urinary phthalates; (f) URXP01 (1-hydroxynaphthalene) and URXP02 (2-hydroxynaphthalene) within urinary polyaromatic hydrocarbons; (g) LBXPFHP (perëuoroheptanoic acid), LBXPFUA (perëuoroundecanoic acid), LBXPFBS (perëuorobutane sulfonic acid), LBXPFSA (perëuorooctane sulfonamide) and LBXPFDO (perëurododecanoic acid) within blood polyfluoroalkyl chemicals; (h) URXOXY (oxypyrimidine) and URXTCC (trans-3-(2,2-dichlorovinyl)-2,2-dimethylcyclopropane carboxylic acid) within Urinary pesticides. The remaining chemical contaminants represented by codes on this table could be found on the official website of NHANES.

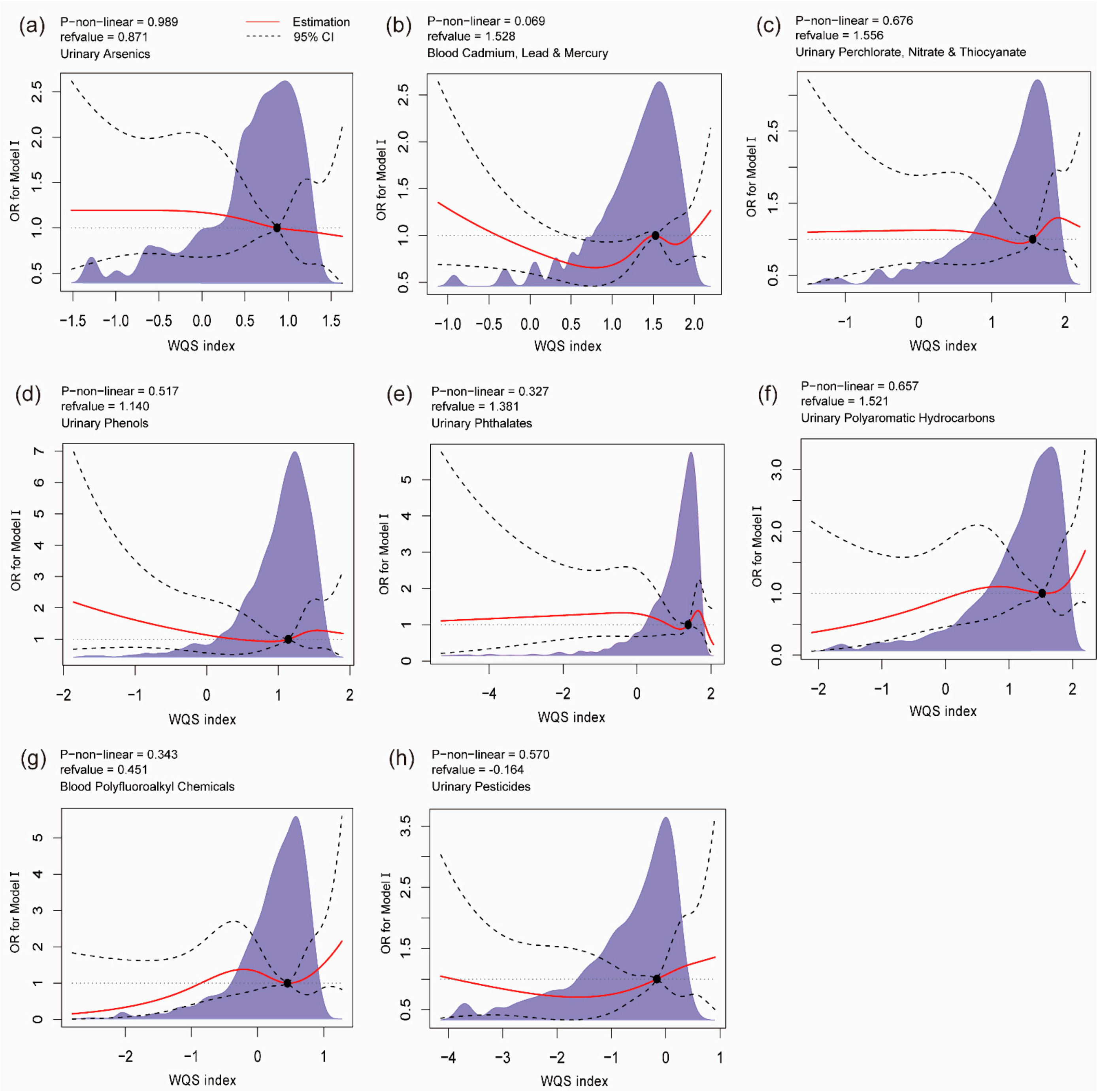

Figure S3 Restricted spline curves for correlations between environmental contaminant mixture groups (WQS indexes) and psoriasis prevalence (Model I cohort). (a) Arsenic species in urine; (b) Cadmium, lead and mercury in blood; (c) Perchlorate, nitrate and thiocyanate in urine; (d) Phenols in urine; (e) Phthalates in urine; (f) Polyaromatic hydrocarbons in urine; (g) Polyfluoroalkyl chemicals in blood; (h) Pesticides in urine. The shaded areas depict data distribution, while the dotted lines correspond to the 95% confidence intervals. Model I was adjusted for age, sex, race, marital status, education, employment, family income-to-poverty ratio, health insurance, BMI, cigarette smoking, alcohol consumption, HEI-2015 and LTPA.

Table S1 Types of environmental contaminant mixture groups included in the weighted quantile sum regressions.

Contaminant mixture groups	Contaminants	Years
Urinary arsenic species (n=7)	Total arsenic, Arsenous acid, Arsenic acid, Arsenobetaine, Arsenocholine, Dimethylarsinic acid, Monomethylarsonic acid	2003-2006, 2009-2014
Blood cadmium, lead and mercury (n=3)	Cadmium, Lead, Mercury	2003-2006, 2009-2014
Urinary perchlorate, nitrate and thiocyanate (n=3)	Perchlorate, Nitrate, Thiocyanate	2005-2006, 2009-2014
Urinary phenols (n=7)	4-tert-octylphenol, Benzophenone-3, Triclosan, Butyl paraben, Ethyl paraben, Methyl paraben, Propyl paraben	2005-2006, 2009-2012
Urinary phthalates (n=13)	Mono(carboxynonyl) phthalate, Mono(carboxyoctyl) Phthalate, Mono-2-ethyl-5-carboxypentyl phthalate, Mono-n-butyl phthalate, Mono-(3-carboxypropyl) phthalate, Mono-ethyl phthalate, Mono-(2-ethyl-5-hydroxyhexyl) phthalate, Mono-(2-ethyl)-hexyl phthalate, Mono-n-methyl phthalate, Mono-isononyl phthalate, Mono-(2-ethyl-5-oxohexyl) phthalate, Mono-benzyl phthalate, Mono-isobutyl phthalate	2005-2006, 2009-2012
Urinary polyaromatic hydrocarbons (n=9)	1-hydroxynaphthalene, 2-hydroxynaphthalene, 3-hydroxyfluorene, 2-hydroxyfluorene, 3-hydroxyphenanthrene, 1-hydroxyphenanthrene, 2-hydroxyphenanthrene, 1-hydroxypyrene, 9-hydroxyfluorene	2003-2006, 2009-2012
Blood polyfluoroalkyl chemicals (n=12)	2-(N-Ethyl-perëuorooctane sulfonamido) acetic acid, Peëuorodecanoic acid, Perëuorooctanoic acid, Perëuorooctane sulfonic acid, Perëuorohexane sulfonic acid, 2-(N-Methyl-perëuorooctane sulfonamido) acetic acid, Perëuorobutane sulfonic acid, Perëuoroheptanoic acid, Perëuorononanoic acid, Perëuorooctane sulfonamide, Perëuoroundecanoic acid, Perëuorododecanoic acid	2003-2006, 2009-2012
Urinary pesticides (n=6)	2,4-D, Paranitrophenol, 4-ëuoro-3-phenoxybenzoic, Trans-3-(2,2-dichlorovinyl)-2,2-dimethylcyclopropane carboxylic acid, 3-phenoxybenzoic, Oxypyrimidine	2009-2014

Table S2 Characteristics of participants in Model II cohort from NHANES 2003-2006 and 2009-2014 cycles.

Variable	Participants[a] with psoriasis n=445 (2.7%)	Participants without psoriasis n=15876 (97.3%)	P-value[b]
Age, years	49.5 ± 16.2	46.4 ± 17.2	<0.001
Sex			0.947
Male	218 (49.0%)	7803 (49.1%)	
Female	227 (51.0%)	8073 (50.9%)	
Race			<0.001
Non-Hispanic White	274 (61.6%)	7507 (47.3%)	
Non-Hispanic Black	60 (13.5%)	3406 (21.5%)	
Other Hispanic	37 (8.3%)	1257 (7.9%)	
Mexican American	35 (7.9%)	2198 (13.8%)	
Other Races	39 (8.8%)	1508 (9.5%)	
Marital status			0.06
Married/cohabited	264 (59.3%)	9488 (59.8%)	
Divorced/separated	76 (17.1%)	2161 (13.6%)	
Widowed	31 (7.0%)	978 (6.2%)	
Unmarried	74 (16.6%)	3249 (20.5%)	
Education			0.499
Less than 9th grade	26 (5.8%)	1180 (7.4%)	
9-12th grade or equivalent	51 (11.5%)	2090 (13.2%)	
High school graduate or GED	103 (23.1%)	3509 (22.1%)	
Some college or associates degree	142 (31.9%)	5034 (31.7%)	
College graduate or above	123 (27.6%)	4063 (25.6%)	
Employment			0.008
Employed	241 (54.2%)	9591 (60.4%)	
Unemployed	204 (45.8%)	6285 (39.6%)	
Family income-to-poverty ratio	2.6 ± 1.7	2.6 ± 1.7	0.992
Health insurance			0.255
Insured	354 (79.6%)	12266 (77.3%)	
Uninsured	91 (20.4%)	3610 (22.7%)	
BMI, kg/m^2	30.0 ± 6.9	29.0 ± 6.9	<0.001
Cigarette smoking	9.0 ± 13.7	5.8 ± 10.4	<0.001
Alcohol consumption	0.5 ± 1.2	0.5 ± 1.1	0.931
Healthy eating index	53.8 ± 12.9	53.2 ± 13.4	0.416
Physical activity	1070.8 ± 2124.5	1259.1 ± 2146.3	0.016
Comorbidities			
Arthritis	184 (41.3%)	3670 (23.1%)	<0.001
Hypertension	195 (43.8%)	5140 (32.4%)	<0.001
Diabetes	78 (17.5%)	1995 (12.6%)	0.002
Cancer	62 (13.9%)	1327 (8.4%)	<0.001
Cardiovascular disease[c]	52 (11.7%)	1035 (6.5%)	<0.001
Stroke	16 (3.6%)	464 (2.9%)	0.407

a: Values were presented as unweighted number (percentage) unless otherwise stated.

b: Continuous variables were compared by Kruskal Wallis rank sum tests and categorical variables were compared using the $\chi 2$ tests.

c: Cardiovascular disease were defined as including congestive heart failure, coronary heart disease, heart attack (myocardial infarction) and angina.
